# Determinants of skeletal muscle loss during initial treatment in patients with ovarian cancer: a brief report

**DOI:** 10.3389/fonc.2026.1794363

**Published:** 2026-03-17

**Authors:** Naomi Nakayama, Daisaku Asai, Tomoka Ishibashi, Tsubasa Maejima, Masako Ishikawa, Kentaro Nakayama

**Affiliations:** 1 Department of General Medicine, East Medical Center, School of Medicine, Nagoya City University, Nagoya, Japan; 2 Department of Obstetrics and Gynecology, East Medical Center, School of Medicine, Nagoya City University, Nagoya, Japan; 3 Department of Obstetrics and Gynecology, School of Medicine, Shimane University, Izumo, Japan

**Keywords:** body composition, debulking surgery and chemotherapy, ovarian cancer, sarcopenia, skeletal muscle loss

## Abstract

**Background:**

We previously reported that skeletal muscle loss during initial treatment is associated with poor outcomes in ovarian cancer. However, its clinical determinants remain incompletely understood.

**Methods:**

We analyzed a previously characterized cohort of patients with ovarian cancer to identify clinical and inflammatory factors associated with skeletal muscle loss during initial treatment. Univariate and multivariate logistic regression analyses were performed using clinical variables, inflammatory markers, and tumor-related factors.

**Results:**

In univariate analysis, elevated neutrophil-to-lymphocyte ratio and the presence of residual tumor were associated with skeletal muscle loss. However, after multivariate adjustment, no variable remained independently associated with muscle loss, and no single clinical or laboratory parameter emerged as a significant predictor.

**Conclusion:**

No independent biomarker predicting early skeletal muscle loss was identified. These findings suggest that muscle loss during initial treatment reflects the overall burden of disease rather than isolated inflammatory or tumor-related factors. Tumor-driven metabolic alterations may contribute to this process and warrant further investigation.

## Introduction

Skeletal muscle loss is increasingly recognized as a clinically important manifestation of cancer-associated systemic deterioration and is associated with impaired treatment tolerance and poor survival in patients with ovarian cancer.

In our previous studies, we demonstrated that skeletal muscle loss occurring during primary debulking surgery and chemotherapy is a strong and independent predictor of poor long-term survival in patients with ovarian cancer, whereas baseline sarcopenic factors assessed at diagnosis were not associated with prognosis (1, 2). These findings indicate that treatment-related skeletal muscle loss represents a dynamic and clinically critical phenotype that emerges during active treatment, rather than a static patient characteristic present at diagnosis.

Subsequently, similar findings have been reported by independent groups across different treatment settings and populations. Loss of skeletal muscle during neoadjuvant chemotherapy has been associated with decreased survival in ovarian cancer patients (3), and muscle loss during primary debulking surgery followed by adjuvant chemotherapy has also been shown to predict poor outcomes in advanced-stage disease (4). Together, these studies establish skeletal muscle loss during active treatment as a robust prognostic marker in ovarian cancer across institutions and therapeutic contexts.

Despite its clear prognostic relevance, the clinical determinants of skeletal muscle loss occurring during primary debulking surgery and chemotherapy remain incompletely understood. Systemic inflammation and tumor burden have been proposed as major contributors to cancer-associated muscle wasting. Recent evidence further supports the close association between tumor microenvironment–driven inflammation and systemic metabolic alterations contributing to skeletal muscle loss in cancer (5). In ovarian cancer, composite indices reflecting inflammation and nutritional status have been reported to correlate with muscle loss during treatment (4). Similar associations have been described in gastrointestinal malignancies, including gastric, colorectal, and pancreatic cancers (6–11), as well as in other gynecologic cancers such as cervical and endometrial cancer (12–14). These observations suggest that skeletal muscle loss during cancer treatment represents a common phenotype of advanced disease and therapeutic stress across cancer types.

However, baseline body composition at diagnosis does not adequately predict subsequent skeletal muscle loss in ovarian cancer. In our previous analyses, body-composition parameters measured at diagnosis failed to predict muscle loss during treatment, and baseline sarcopenia was not associated with long-term outcomes (1, 2). These findings suggest that skeletal muscle wasting in ovarian cancer is not a pre-existing vulnerability but rather a dynamic process that develops during treatment, reflecting complex interactions between tumor burden, treatment-related stress, and host response.

Beyond systemic inflammation and tumor burden, cancer-associated skeletal muscle loss is increasingly recognized as a multifactorial process that cannot be fully explained by isolated clinical or laboratory markers (15, 16). Tumor–host interactions and systemic metabolic stress may contribute to this process, and dynamic changes in skeletal muscle may represent an integrated manifestation of disease-related physiological burden that evolves during cancer treatment (17, 18). Accordingly, it remains unclear whether commonly used clinical variables and inflammatory markers independently predict skeletal muscle loss occurring during primary debulking surgery and chemotherapy in patients with ovarian cancer.

Although skeletal muscle loss during treatment has consistently been shown to predict poor survival in ovarian cancer, it remains unclear whether this dynamic phenotype can be explained by identifiable clinical or inflammatory biomarkers. Previous studies have primarily focused on its prognostic significance, whereas determinants of treatment-related muscle loss have not been systematically evaluated using multivariate modeling.

Therefore, the present study aimed to identify independent clinical and inflammatory determinants of skeletal muscle loss during primary debulking surgery and chemotherapy in ovarian cancer patients. To our knowledge, this is the first study specifically designed to evaluate whether treatment-related skeletal muscle loss can be independently predicted by commonly used clinical and laboratory parameters in a multivariate framework.

## Methods

### Patients

The patient cohort analyzed in this study was derived from a previously published dataset (2), which investigated the prognostic impact of muscle and fat loss in ovarian cancer. In contrast, the present analysis was designed to identify clinical and inflammatory determinants of skeletal muscle volume change during primary debulking surgery and adjuvant chemotherapy.

Inclusion criteria were: (1) histologically confirmed epithelial ovarian cancer; (2) primary debulking surgery followed by adjuvant chemotherapy; and (3) availability of evaluable CT imaging at baseline and after completion of initial treatment.

Exclusion criteria included: (1) inadequate CT imaging for skeletal muscle assessment; (2) missing essential clinical or laboratory data; and (3) incomplete initial treatment.

None of the statistical analyses or numerical results reported here were included in the prior publication.

### Assessment of skeletal muscle volume

Skeletal muscle volume was quantified using computed tomography (CT) images obtained at baseline and after initial treatment. Cross-sectional muscle area was measured at the third lumbar vertebra (L3) using a validated image analysis method as previously described (2). Skeletal muscle index (SMI) was calculated by normalizing muscle area to height squared (cm²/m²). Muscle volume change was defined as the relative change in SMI between the two time points as previously described (2).

### Definition of muscle loss

Patients were classified into those with and without significant muscle loss according to predefined criteria based on SMI change during initial treatment as reported previously (2).

### Clinical and laboratory variables

The following variables were evaluated as potential determinants of skeletal muscle loss: age, FIGO stage, neutrophil-to-lymphocyte ratio (NLR), C-reactive protein (CRP), pre-existing comorbidities, including diabetes mellitus and a history of deep vein thrombosis, perioperative complications, residual tumor after primary debulking surgery, and ascites cytology. CRP was dichotomized at 0.14 mg/dL, corresponding to the upper limit of normal defined by our institutional laboratory standards. Perioperative complications were included as a candidate variable because postoperative morbidity may reflect surgical stress, inflammatory burden, and impaired nutritional intake, all of which are potential contributors to treatment-related skeletal muscle loss. Perioperative complications were defined as clinically relevant postoperative adverse events (Clavien–Dindo grade ≥II) occurring within 30 days after primary debulking surgery, including infectious, gastrointestinal, or systemic complications requiring pharmacological or invasive intervention. Age was included as a biologically relevant covariate because advancing age is a well-established determinant of skeletal muscle decline. Age was analyzed as a categorical variable and dichotomized at the median value of the cohort to ensure balanced group sizes and model stability. FIGO stage was categorized as stage IV versus non–stage IV, as stage IV disease represents a distinct clinical entity characterized by overt distant metastasis and a substantially higher systemic disease burden compared with other earlier stages. The NLR was dichotomized using a predefined cutoff value of ≥3, as this threshold has been widely used in previous oncologic studies to distinguish patients with heightened systemic inflammatory status. Variables known to be associated with prognosis, such as age and residual tumor status, were included *a priori* as candidate predictors of muscle loss.

### Statistical analysis

Data were statistically analyzed using SPSS version 27.0 (IBM Corporation, Armonk, NY, USA). Univariate logistic regression analysis was performed to identify factors associated with skeletal muscle loss during initial treatment. Odds ratios (ORs) and 95% confidence intervals (CIs) were calculated. Variables showing statistical significance (p < 0.05) in univariate analysis were entered into the multivariate logistic regression model to identify independent predictors. Given the limited sample size, the number of variables included in the multivariate model was restricted to minimize the risk of overfitting. A two-sided p value <0.05 was considered statistically significant. Because this study was a retrospective secondary analysis of an existing cohort, no formal sample size calculation was performed prior to the analysis. The sample size was determined by the number of eligible patients in the original dataset.

## Results

Baseline characteristics of the study cohort are summarized in **Table 1**.

**Table 1:** Baseline characteristics of the study cohort (n = 58).

Variable	Value
Body mass index (kg/m²)	22.73 ± 3.82
Age (years)	60.82 ± 12.71
Performance status	0.08 ± 0.38
Hospitalization period (days)	15.5 (10.3)
Days between CT scans (days)	225.0 (80.5)
Histology, n (%)	
Serous	23 (39.7)
Endometrioid	14 (24.1)
Mucinous	9 (15.5)
Clear cell	12 (20.7)
FIGO stage, n (%)	
I	25 (43.1)
II	7 (12.0)
III	17 (29.3)
IV	9 (15.6)

A total of 58 patients were included in the analysis. Among them, 32 patients (55.2%) were classified as having skeletal muscle loss during initial treatment.

In univariate logistic regression analysis (**Table 2**), an elevated neutrophil-to-lymphocyte ratio (NLR ≥3) and the presence of residual tumor were significantly associated with skeletal muscle loss. Elevated C-reactive protein levels and a history of deep vein thrombosis showed borderline associations, whereas FIGO stage IV disease was not significantly associated.

**Table 2:** Data are presented as mean ± standard deviation (SD), median (interquartile range, IQR), or number (%), as appropriate.

Variable	Odds Ratio (OR)	95% CI	p value
Age >61 years	2.941	0.935–9.256	0.065
FIGO stage IV	3.360	0.634–17.819	0.154
NLR ≥3 at diagnosis	3.490	1.147–10.621	0.028
CRP ≥0.14 mg/dL	2.567	0.877–7.512	0.085
Diabetes mellitus	1.909	0.559–6.524	0.302
Deep vein thrombosis	4.696	0.915–24.097	0.064
Perioperative complications	0.933	0.270–3.222	0.913
Residual tumor	3.375	1.062–10.711	0.039
Ascites cytology positive	1.597	0.530–4.816	0.406
Bevacizumab use	1.095	0.222–5.399	0.911

Odds ratios (ORs) and 95% confidence intervals (CIs) were calculated using logistic regression. Perioperative complications were defined as postoperative adverse events of Clavien–Dindo grade ≥II occurring within 30 days after surgery. CRP was dichotomized at 0.14 mg/dL (upper limit of normal). DM, diabetes mellitus.

Univariate logistic regression analysis of factors associated with skeletal muscle loss during initial treatment.

In the multivariate logistic regression model, none of the variables, including NLR and residual tumor status, remained independently associated with skeletal muscle loss (**Table 3**).

**Table 3:** Multivariate logistic regression analysis of factors associated with skeletal muscle loss during initial treatment.

Variable	Adjusted OR	95% CI	p value
NLR ≥3 at diagnosis	2.151	0.606–7.634	0.236
Residual tumor	3.589	0.951–13.543	0.059

Variables showing statistical significance (p < 0.05) in univariate analysis were entered into the multivariate logistic regression model. The number of variables included in the multivariate model was restricted to minimize the risk of overfitting given the limited sample size.

These results indicate that although inflammatory and tumor-related factors were associated with skeletal muscle loss in univariate analyses, no single variable demonstrated independent predictive value after multivariate adjustment.

## Discussion

In this brief report, we evaluated clinical and inflammatory determinants of skeletal muscle loss during initial treatment in patients with ovarian cancer. Although elevated neutrophil-to-lymphocyte ratio (NLR) and the presence of residual tumor were associated with muscle loss in univariate analysis, these associations disappeared after multivariate adjustment, indicating that they are not independent predictors of early muscle wasting. Perioperative complications were included as a surrogate for surgical stress and postoperative inflammatory burden; however, they were not independently associated with skeletal muscle loss in the multivariate analysis. This finding further supports the notion that early muscle wasting during initial treatment reflects an integrated physiological burden of disease and therapy rather than the impact of discrete postoperative events. The neutrophil-to-lymphocyte ratio (NLR) is a widely used marker of systemic inflammation reflecting the balance between innate immune activation and adaptive immune suppression, and elevated NLR has been associated with skeletal muscle loss and sarcopenia during treatment in several malignancies, including gastrointestinal and lung cancers (19, 20).

These findings are consistent with and extend previous reports in ovarian cancer. Rutten et al. demonstrated that skeletal muscle loss during neoadjuvant chemotherapy (NACT), rather than baseline muscle mass, was strongly associated with poor survival (3). In a different treatment context, Huang et al. reported that skeletal muscle loss occurring during primary debulking surgery followed by adjuvant chemotherapy was associated with composite indices reflecting systemic inflammation and nutritional status (4). Together, these studies suggest that muscle wasting during treatment reflects the global physiological stress imposed by advanced disease and its therapy, rather than the effect of a single biological pathway.

An important context for the present findings is our previous work demonstrating that baseline sarcopenic factors at diagnosis were not associated with long-term outcomes in ovarian cancer patients (1). In contrast, we subsequently showed that skeletal muscle loss occurring during primary debulking surgery and chemotherapy was strongly associated with poor prognosis (2). Moreover, in that cohort, baseline body-composition parameters did not predict subsequent muscle loss. Taken together, these findings indicate that muscle wasting in ovarian cancer is not a static patient characteristic present at diagnosis, but rather a dynamic host response that emerges during treatment and reflects tumor–host metabolic stress. The present analysis further extends this concept by demonstrating that this dynamic muscle loss cannot be explained by single inflammatory or tumor-burden biomarkers.

In addition to inflammatory and tumor-related factors, we included comorbid conditions as candidate variables in the analysis. Diabetes mellitus was considered because pre-existing diabetes has been reported to exacerbate cancer-associated cachexia and systemic inflammation, and insulin resistance is recognized as a mechanistic contributor to skeletal muscle wasting in cancer patients (21, 22). We also included a history of deep vein thrombosis (DVT) as a clinical covariate. Although DVT itself is not known to directly induce muscle loss, previous studies in oncology populations have demonstrated associations between low skeletal muscle mass and venous thromboembolism events, supporting the inclusion of DVT as a relevant comorbidity reflecting systemic vulnerability (23, 24). However, in the present cohort, neither diabetes mellitus nor DVT was associated with skeletal muscle loss in univariate or multivariate analyses, suggesting that these comorbidities do not independently drive early muscle wasting during initial treatment in ovarian cancer.

In addition to comorbidities, we included exposure to bevacizumab as a treatment-related variable in the present analysis. Bevacizumab is a monoclonal antibody targeting vascular endothelial growth factor (VEGF), and VEGF signaling plays an important role not only in tumor angiogenesis but also in physiological processes relevant to skeletal muscle, including microvascular maintenance, perfusion, metabolic adaptation, and muscle regeneration. Recent bioinformatic analyses have suggested a potential association between bevacizumab and the expression of muscle atrophy–related gene signatures, raising the possibility of treatment-related muscle toxicity (25). Moreover, clinical studies in metastatic colorectal cancer have reported longitudinal skeletal muscle loss during bevacizumab-containing chemotherapy, as assessed by serial computed tomography (26). Based on these mechanistic and clinical considerations, bevacizumab exposure was included as a candidate variable in our analysis. However, in the present cohort of patients with ovarian cancer, bevacizumab use was not associated with skeletal muscle loss in either univariate or multivariate analyses, suggesting that bevacizumab itself is unlikely to be a major determinant of early muscle wasting in this setting.

Evidence from gastrointestinal malignancies further supports this concept. In gastric cancer, profiles combining skeletal muscle atrophy with inflammatory markers such as NLR stratify prognosis (6), and muscle loss during chemotherapy is associated with poor survival (7). In colorectal cancer, longitudinal skeletal muscle loss during systemic therapy independently predicts adverse outcomes and is associated with peri-operative clinicopathological characteristics (8, 9). In pancreatic cancer, early skeletal muscle loss during first-line chemotherapy is common and prognostically unfavorable, even when supportive interventions such as physical activity are provided (10, 11). Across these malignancies, muscle loss represents a robust phenotype of cancer-associated systemic deterioration rather than a tumor-specific phenomenon.

A similar pattern has been observed in other gynecologic malignancies. In cervical cancer treated with definitive chemoradiotherapy, skeletal muscle loss during treatment is a strong imaging biomarker of survival, and volumetric analyses confirm that dynamic body composition changes provide prognostic information beyond baseline sarcopenia (12, 13). In advanced endometrial cancer, decline in skeletal muscle radiodensity during therapy—reflecting deterioration in muscle quality—predicts poor survival even when body weight or BMI remains stable (14). These findings underscore that clinically relevant muscle deterioration may be occult and not captured by conventional anthropometric or laboratory measures.

Beyond inflammation and tumor burden, accumulating evidence suggests that cancer-associated muscle loss is driven by tumor–host metabolic interactions. Tumors reprogram intracellular metabolism to survive and grow under nutrient-limited conditions, engaging alternative pathways including gluconeogenic flux, amino acid utilization, and serine/glycine biosynthesis (15–18). Stable isotope tracing and metabolic flux studies have emerged as powerful tools to map these metabolic adaptations *in vivo*, revealing that tumors dynamically utilize and compete for circulating metabolites such as glutamine and alanine, which may be derived from host tissue breakdown (15, 16). Furthermore, amino acid metabolism has been implicated in both cancer cell growth and cancer-associated cachexia, suggesting that dysregulation of circulating amino acids contributes to host tissue wasting under tumor metabolic demand (17). Recent spatial isotope deep tracing approaches further support the concept that tumors can exert inter-organ metabolic influence that contributes to cachexia phenotypes (18).

In ovarian cancer, we recently demonstrated that NAC1 transcriptionally regulates mitochondrial phosphoenolpyruvate carboxykinase (PCK2) and activates truncated gluconeogenesis, conferring a growth advantage under metabolic stress (27). Such tumor-intrinsic metabolic programs provide a mechanistic basis for substrate competition between tumor and host muscle, offering an explanation for why no single inflammatory or tumor-burden biomarker emerged as an independent predictor in our multivariate model.

Although our study did not directly measure circulating metabolites or tumor metabolic markers, prior mechanistic studies provide a biologically plausible framework to interpret our findings. These interpretations remain speculative and require validation in dedicated mechanistic investigations.

Clinically, these findings caution against biomarker-based screening using single laboratory parameters. Across cancer types, muscle loss appears to be a multidimensional manifestation of tumor-driven metabolic remodeling, treatment stress, and host response. Integrating body-composition imaging with metabolic and clinical indices may therefore be required to identify patients who would benefit from early nutritional and supportive interventions.

## Limitations

Several limitations should be acknowledged. First, this was a retrospective, single-center secondary analysis with a limited sample size, which may have reduced power to detect modest associations. Given the limited sample size, the study was powered to detect only relatively strong associations, and moderate associations may have gone undetected. Therefore, the absence of independent predictors should be interpreted cautiously. Second, we did not directly measure tumor metabolic activity or circulating metabolites, precluding definitive mechanistic conclusions regarding metabolic drivers of muscle loss. Third, skeletal muscle change was assessed at two time points during initial treatment; more frequent longitudinal assessments could better capture the dynamics of muscle wasting. Fourth, the relatively small number of patients in each residual tumor category (R0/R1/R2) precluded detailed stratified or interaction analyses, which should be addressed in larger future studies. Finally, because the cohort was derived from previously reported datasets, external validation in independent populations is required to confirm generalizability.

## Data Availability

The dataset contains sensitive clinical and imaging data from human participants and cannot be made publicly available due to ethical and privacy restrictions approved by the institutional review board. Requests to access the datasets should be directed to NN, emc6079@med.nagoya-cu.ac.jp.
